# Geometric morphometric analysis in female freshwater crabs of Sarawak (Borneo) permits addressing taxonomy-related problems

**DOI:** 10.7717/peerj.6205

**Published:** 2019-02-14

**Authors:** Jongkar Grinang, Indraneil Das, Peter K.L. Ng

**Affiliations:** 1 Institute of Biodiversity and Environmental Conservation, Universiti Malaysia Sarawak, Kota Samarahan, Sarawak, Malaysia; 2 Lee Kong Chian Natural History Museum, National University of Singapore, Singapore, Singapore

**Keywords:** Freshwater crab, Geometric morphometrics, *Isolapotamon*, Taxonomy

## Abstract

The taxonomy of freshwater crabs requires a paradigm change in methodological approaches, particularly in investigations that use morphological techniques. The traditional morphometric approach (two-dimensional measurements) tends to be inappropriate for the identification of freshwater crabs due to their variable external morphology and lack of gonopods (conventionally used for the identification of male crabs) in females. In this study, we explore the potential use of the geometric morphometric technique for identification of female freshwater crabs, and identify taxonomic key characteristics of species. The shape of the carapace could be a good characteristic for the identification of female crabs, especially when the geometric morphometric technique is used. It was observed that the shape of the carapace has an advantage over the shape of the pleon and chela because its relatively flat orientation allows more consistent and easier data preparation for geometric morphometric analysis. The geometric morphometric technique is inexpensive, relatively less time consuming to employ, and accurate. This technique is convenient when dissection to examine the gonopods is not possible, which can damage the specimen in the case of endangered or rare species. Since the technique was used herein for only two species, more compelling and extensive evidence is needed before the reliability of the method can be proven.

## Introduction

Geometric morphometrics is a rapidly evolving technique within the vast field of traditional morphometrics (Rohlf & Marcus, 1993; Marcus & Corti, 1996; Zelditch et al., 2004). The technique works on the hypothesis that landmarks on the body of an organism carry unique characteristics of the species (Zelditch et al., 2004). The three-dimensional approach is hailed as a revolution in morphometrics for its paradigm shift away from visualizing the results in terms of scatter plots (Adams, Rohlf & Slice, 2004). It has been successfully employed to discriminate variation among species and populations in a number of animal groups, including insects (Sadeghi, Adriaens & Dumont, 2009; Tüzün, 2009), water mites (Becerra & García-Valdecasas, 2004), lizards (Kaliontzopoulu, Carretero & Llorente, 2007), marine crabs (Rosenberg, 2002; Rufino, Abelló & Yule, 2004; Ledesma, Van Der Molen & Barón, 2010; Hampton et al., 2014), fishes (Chakrabarty et al., 2010), and small mammals (Loy, Corti & Marcus, 1993; Abiadh et al., 2010). Nonetheless, examples of the application of the technique in freshwater crabs are lacking, particularly in south-east Asia. The technique potentially faces challenges from ontogenetic shape changes and character displacement in addition to cryptic speciation.

One of the major challenges in studying freshwater crabs is their identification, which currently relies mainly on the structure of the male reproductive organs. Female crabs were traditionally not preferred for species description as they lack gonopods. However, no study appeared to investigate taxonomically informative characteristics in female crabs that could be used for species description and identification, useful for species delineation, taxonomy, and identification related to biodiversity inventories and understanding the evolution of secondary sexual dimorphism within lineages. Descriptions of male crabs were traditionally made based on two-dimensional morphological characteristics, particularly the gonopods (Ng, 1988). The problem in the identification of female crabs, especially at the species level, thus remains unresolved. The challenges indicate there is need for a new approach. The standard two-dimensional measurements of length and width are rarely sufficient for identification of freshwater crabs because the external morphology of the animal tends to be highly variable (Ng, 1988; Cumberlidge, 1999), whereas linear measurements tend to be auto correlated with the size of animals (Zelditch et al., 2004).

The freshwater crabs of Sarawak have been studied since the early 19th century, and are currently represented by 48 species (Ng, Guinot & Davie, 2008). A major challenge in the estimation of the total number of freshwater crabs in Sarawak is the limited identification tools available for the fauna. Identification of freshwater crabs in Sarawak primarily follows the dichotomous keys provided by Ng & Tan (1998) and Ng (2004) that deal with Malaysian families, genera, and selected species such as *Parathelphusa* spp. and *Isolapotamon* spp. The taxonomy of the potamid genus *Isolapotamon* (Bott, 1968) is well understood, and seven species are found in Sarawak (Bott, 1968; Ng & Yang, 1986; Ng, 1987; Ng & Tan, 1998). The genus is characterized by a broad carapace, the presence of an epibranchial tooth, and the elongated male first gonopods (Ng & Tan, 1998). Among the Bornean freshwater crabs, species of *Isolapotamon* are much easier to distinguish by the unique structure of the male first gonopod (Ng & Yang, 1986; Ng, 1987; Ng & Tan, 1998). However, accurate species identification is challenging if the collection includes only females and juvenile males. The present study applies geometric morphometric analysis to identify the best characteristic for the diagnosis of female crabs. It is hypothesized that the structure selected for using geometric morphometric analysis will have taxonomic importance if it: (1) shows consistency within the female crabs of a particular species; (2) demonstrates consistent variation among the species; and (3) is easy to visualize and/or measure (Zelditch et al., 2004).

## Materials and Methods

**Figure 1:**
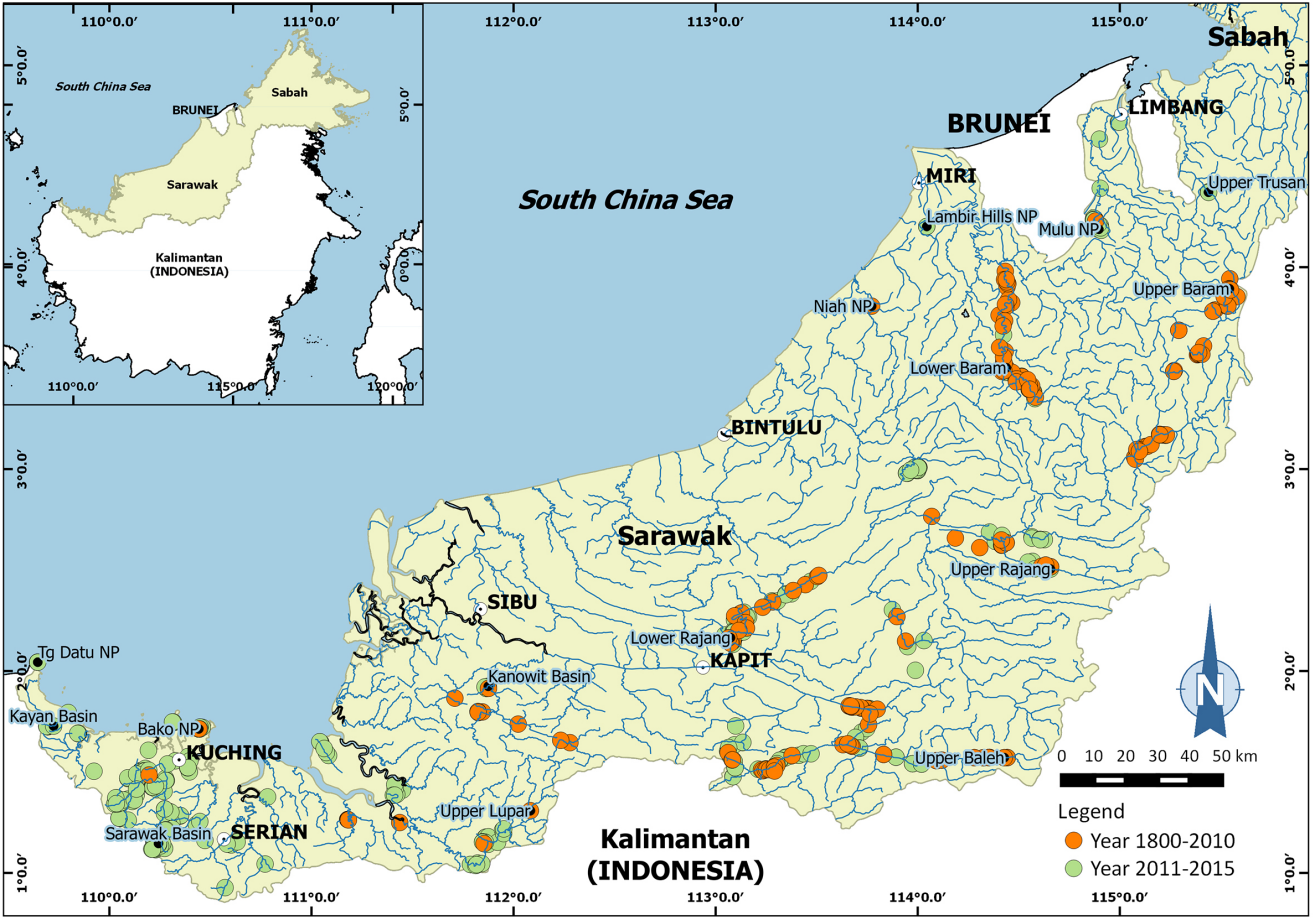
Map showing surveyed areas in Sarawak (Malaysia), Borneo between 1800 and 2015.

**Figure 2:**
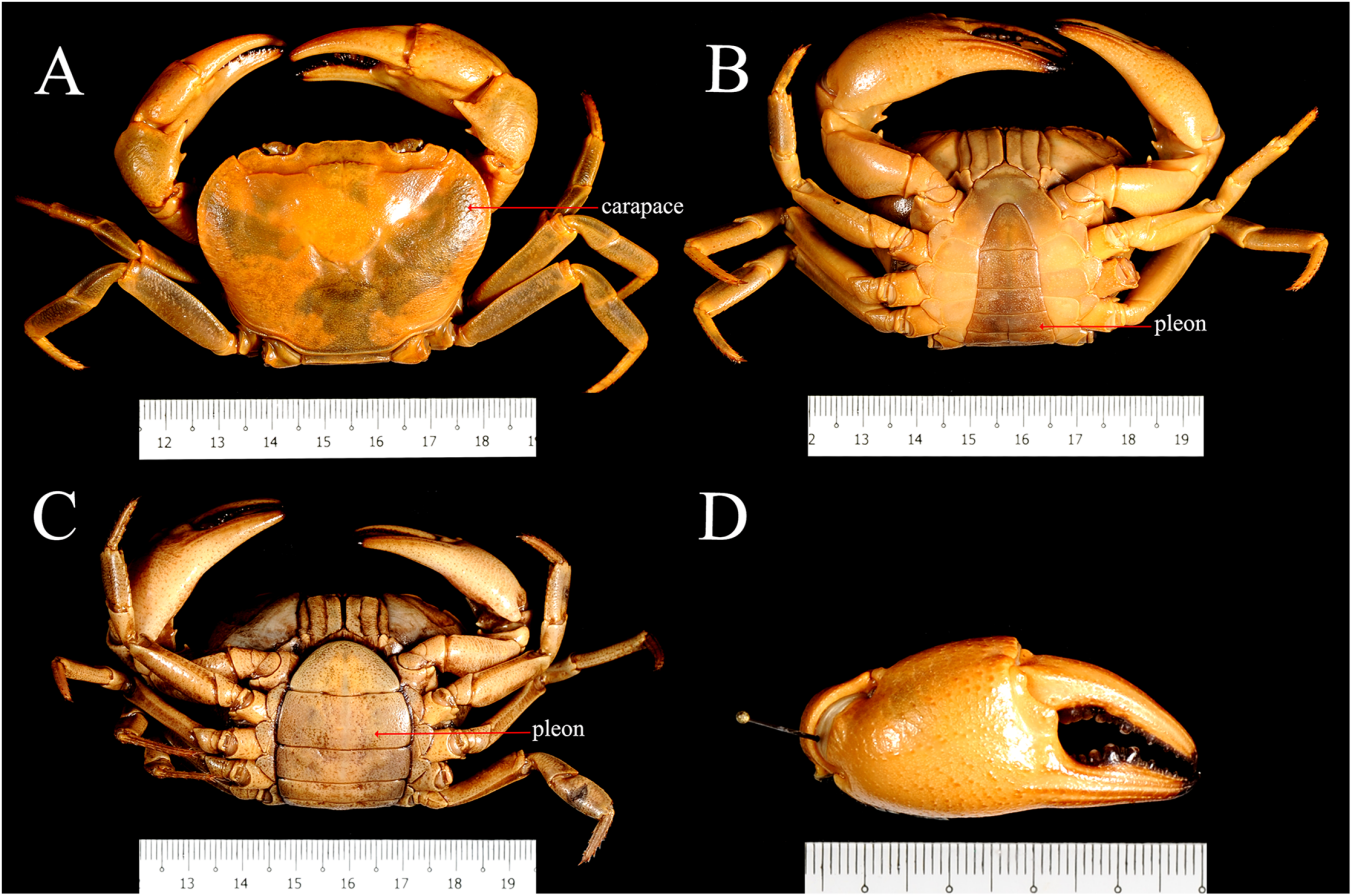
Preparation of images of *I. nimboni* for geometric morphometric analysis. (A) Male carapace; (B) male pleon; (C) female pleon; (D) male right or major chela.

Two species of the genus *Isolapotamon* were selected for geometric morphometric analysis: *I. consobrinum* (De Man, 1899) and *Isolapotamon nimboni* Ng, 1987. The specimens were collected from some major river systems and tributaries in Sarawak (Malaysia), Borneo (Fig. 1). Research permit No. 349/2012 for this study was granted by Forest Department of Sarawak. A total of 25 males and 14 females of *I. consobrinum*, and four males and 10 females of *I. nimboni* were used for geometric morphometric analysis. Crabs were identified following Ng & Tan (1998) and measured for carapace width and carapace length to the nearest 0.01 mm (Table 1). Three morphological characteristics of the crabs were selected for this analysis: carapace, pleon, and right chela. Because chelipeds of the genus *Isolapotamon* have always asymmetrical, only specimens with right major chela were used to minimize potential influences on data analysis. Data preparation involved setting up the specimens and taking images of the morphological characteristics of the crabs. The character was placed horizontally on a black cloth with a scale next to it, which is useful for further analysis (Fig. 2). Digital images of the characters were captured using a Nikon™ D700 DSLR and Tamron™ 90 mm macro lens.

**Table 1:** Measurements (in mm) of *Isolapotamon consobrinum* and *Isolapotamon nimboni*.

Characters	*Isolapotamon consobrinum*		*Isolapotamon nimboni*	
	Male (*n* = 25)	Female (*n* = 14)	Male (*n* = 4)	Female (*n* = 10)
Carapace width	31.3–62.8	23.8–56.5	53.8–58.6	38.5–56.1
Carapace length	20.3–47.4	11.6–40.9	39.7–42.3	28.7–40.7

**Figure 3:**
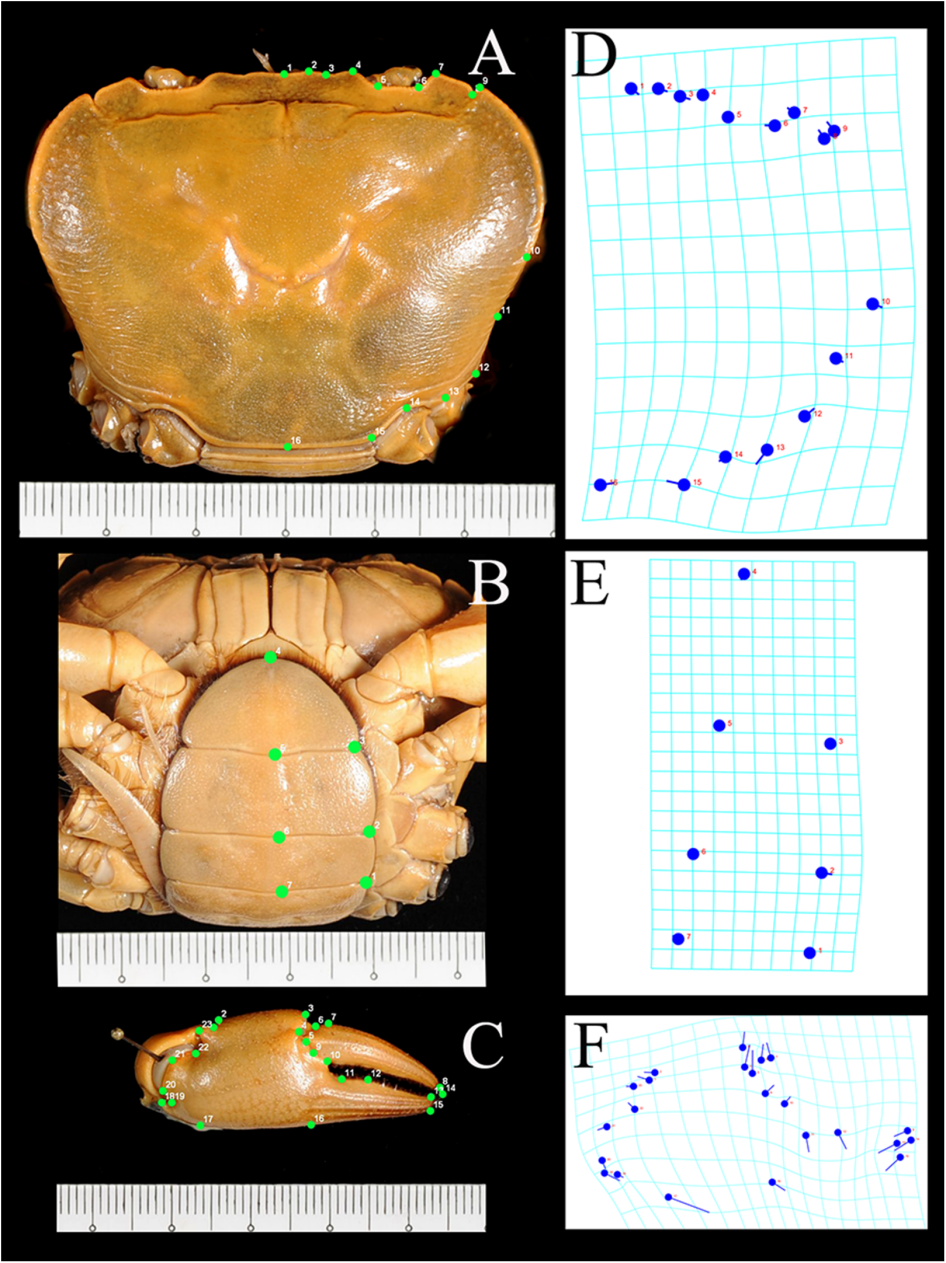
Landmarks on the carapace, pleon, and chela of *I. nimboni* identified for geometric morphometric analysis Blue dots are coordinates on grids corresponding to landmarks (green dots) on carapace (A, D), pleon (B, E) and chela (C, F).

Data analysis involved generating landmarks and processing the geometry parameters of the images using *tpsUtil* and *tpsDig* programs (Rohlf, 2004), followed by multivariate analyses such as general Procrustes analysis (GPA) superimposition, principle components analysis (PCA), canonical variate analysis (CVA), and discriminant function analysis (DFA) in the MorphoJ Package (Klingenberg, 2011). Landmarks selected for analysis were those considered best representing the morphology useful for diagnosis. Due to the symmetrical of structures, landmarks for the carapace and pleon were recorded on the right side to avoid duplication of equivalent landmarks and statistical problems (Zelditch et al., 2004). Landmarks for the pleon were obtained from fourth to sixth somites and the telson. Pleonal somites 1–3 were excluded to avoid the effects of image distortion. A total of 16 landmarks were set for the carapace, seven for the pleon, and 23 for the chela (Fig. 3). The *tpsUtil* program was used to build image files of the characters, whereas landmarks on the characters were constructed using the *tpsDig* program. GPA superimposition was used to extract shape information in the form of coordinates (landmarks) and to remove variation unrelated to the form, such as orientation, rotation, and scale (Zelditch et al., 2004). Variations of the coordinates (in the form of covariance) were examined using PCA followed by CVA for simplifying the description of differences between groups. DFA was used to determine whether the groups could be reliably distinguished. Procrustes distances of CVA between groups were tested for significance with the 1,000 permutations procedure.

## Results

The underlying assumption in geometric morphometric analysis is that the shape variations between sex and species are unrelated to orientation, rotation, and scale during the preparation of the samples. The technique thus allows for acceptance of the hypothesis that characters display consistent shape appearance across female crabs of a particular species, and demonstrates that consistent shape variation across a species must be the best characteristic for species diagnosis of female freshwater crabs.

**Figure 4:**
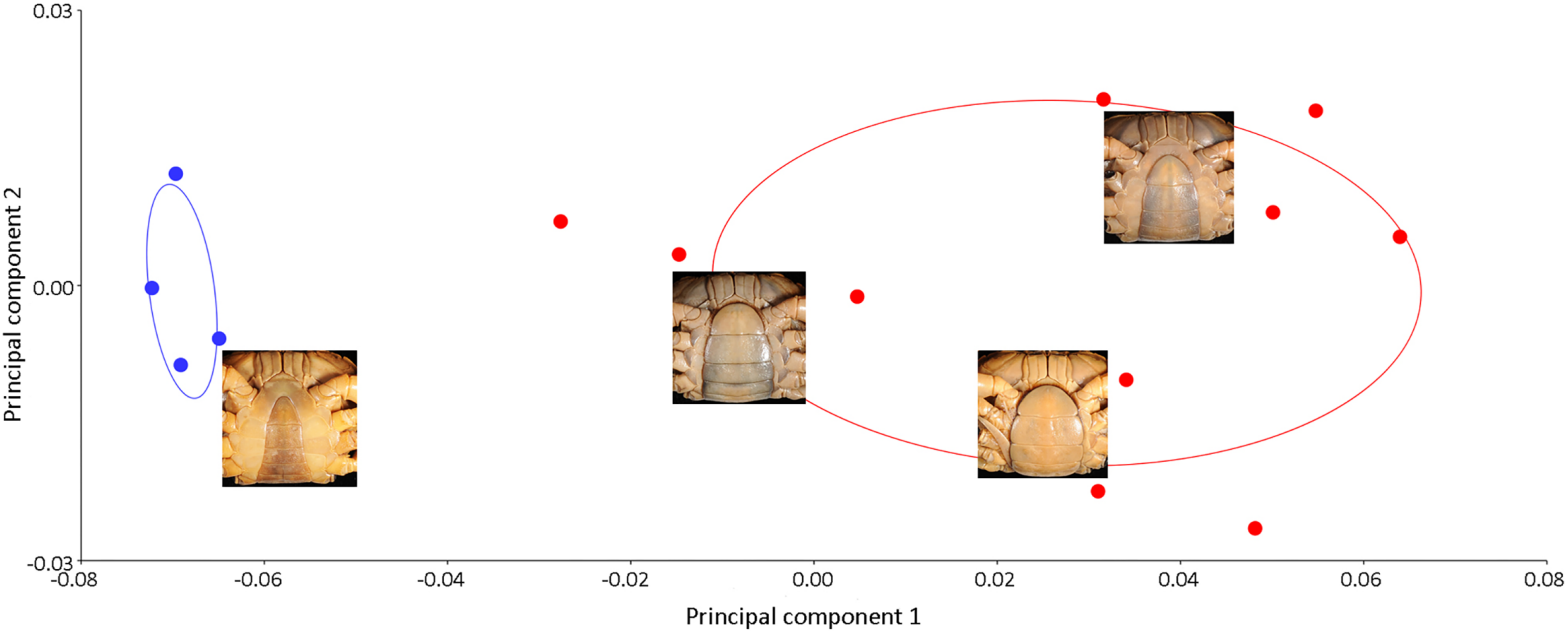
Biplot of PCA showing distinct variations in the shape of the male (blue dots) and female (red dots) pleon of *I. nimboni*. PC1 and PC2 depict 84.4% and 5.99% of variance, respectively. Variations in the shape of the female pleon among different life-history stages: completely mature, bottom right; partially mature, center right; subadult, top right.

**Figure 5:**
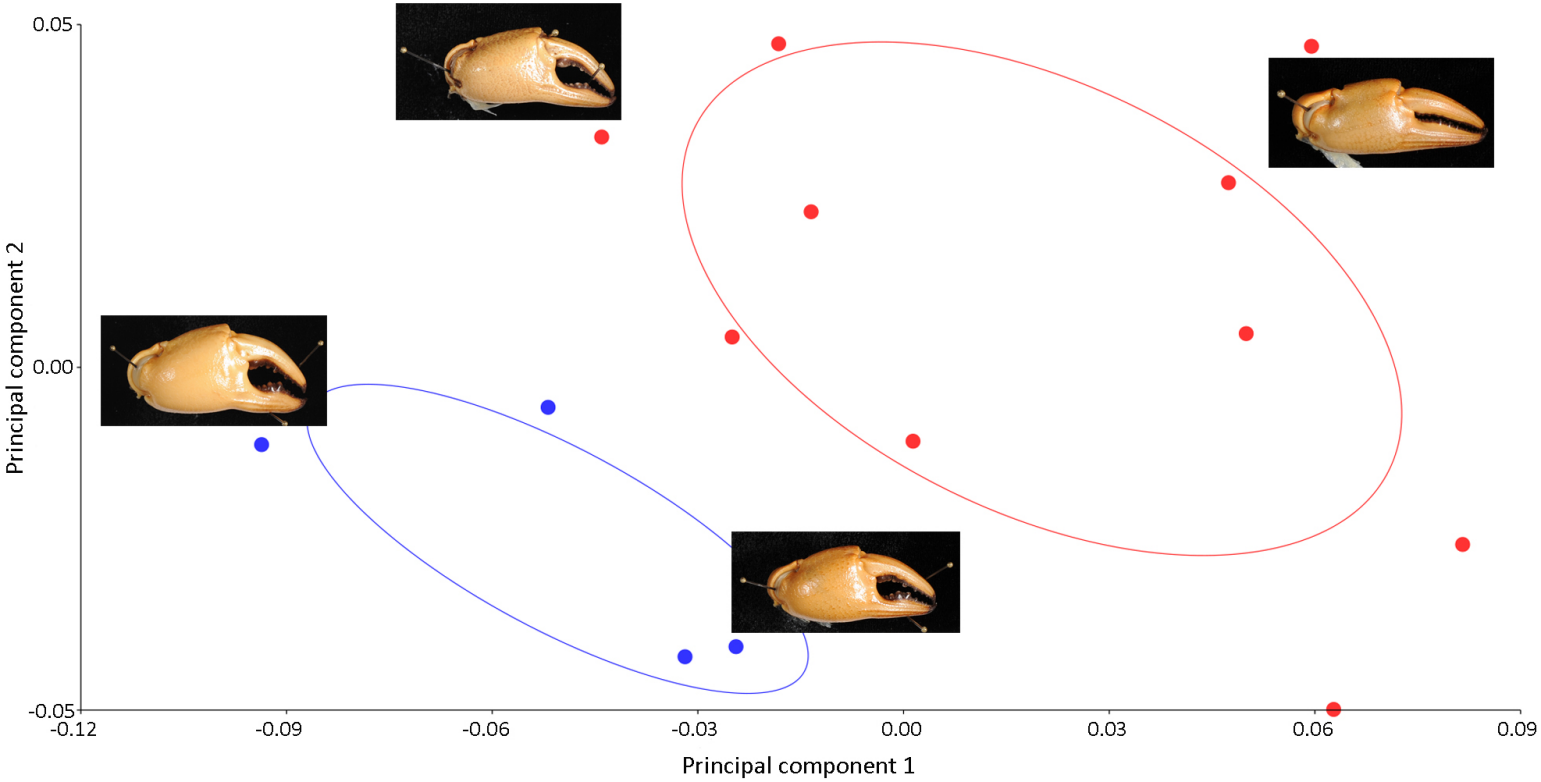
PCA showing significant variations in the shape of the male chela (blue dots) and female chela (red dots) of *I. nimboni*. PC1 and PC2 depict 56.50% and 22.13% of variance, respectively.

Principle components analysis revealed a distinct separation of the shape of the pleon and major chela between male and female crabs of *I. nimboni* (Figs. 4 and 5). Variations in the shape of the pleon among individual female crabs were also observed, which differentiate the following life-history stages: completely mature, partially mature, and subadult (Fig. 4). Alternatively, the age characteristic is insignificant among male crabs regarding the shape of the pleon (Fig. 4). Variations in the shape of major chela among different individuals (both males and females) of *I. nimboni* (Fig. 5) were not clearly related to the crab’s age, but possibly resulted from the regeneration effects and deformity either due to fights or feeding usages of the chela. Results of PCA are supported by both CVA and DFA, at least for the pleon (Table 2). The Procrustes distance of pleon and major chela between the sexes is 0.097 (*p* < 0.0001) and 0.080 (*p* = 0.005), respectively, which provides statistical evidence for the difference in the shape of the two structures between males and females of *I. nimboni*. Results of Hotelling’s *T*^2^ (DFA) indicate that the statistical evidence of differences in the shape of the pleon, as shown by CVA, may have a taxonomic implication, whereas the differences in the shape of major chela could be by chance (Table 2).

**Table 2:** Canonical variate analysis and discriminant function analysis indicating morphological differences in the shape of the pleon and chela between the male and female crabs of *Isolapotamon nimboni*.

Characters	Canonical variate analysis		Discriminant function analysis	
	Procrustes distance	*p*-Value	Hotelling’s *T*^2^	*p*-Value
Pleon	0.097	<0.0001	1,250.79	0.008
Carapace	0.023	0.163	34.36	0.937
Chela	0.080	0.005	69.54	0.824

**Figure 6:**
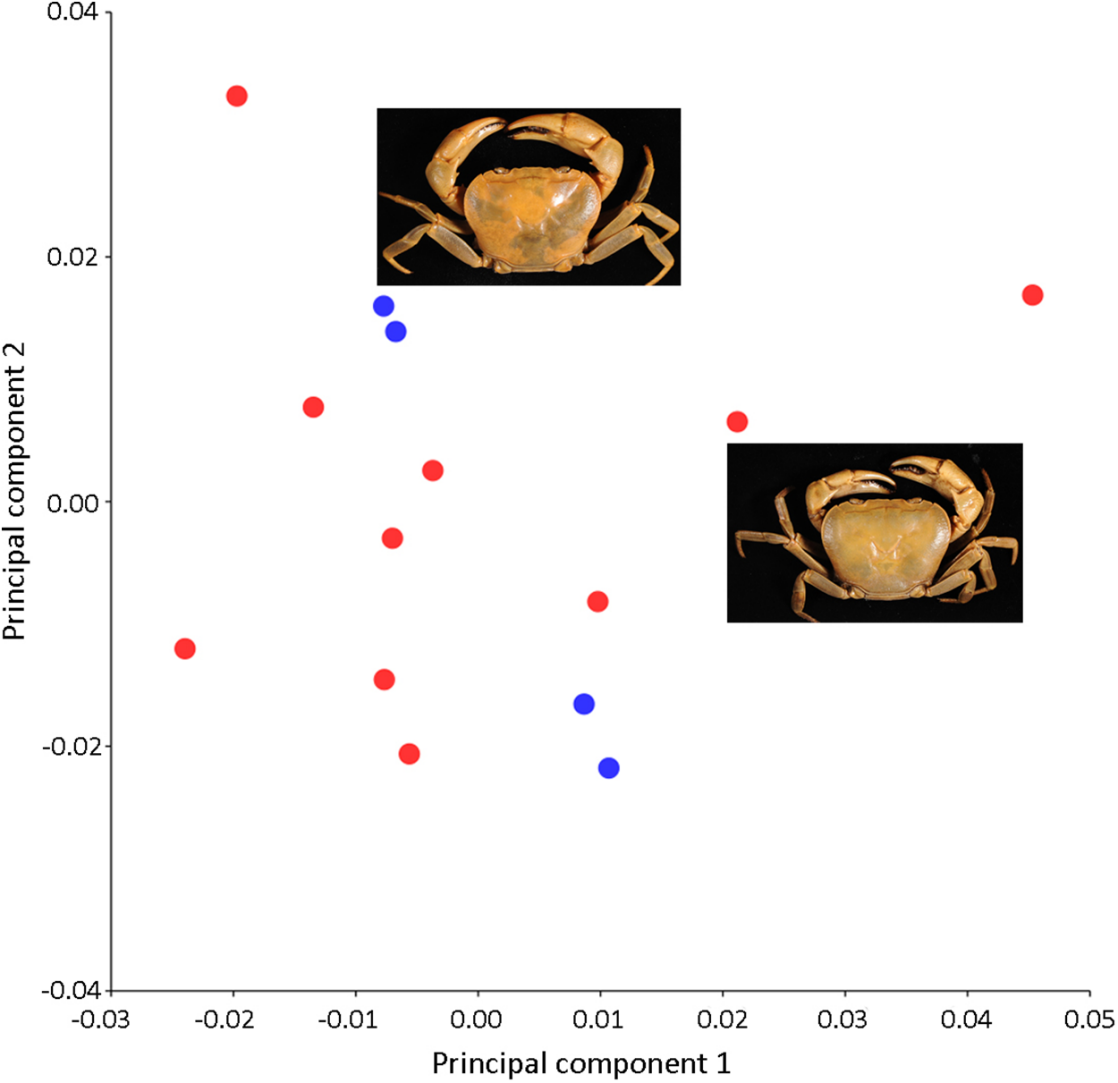
PCA showing insignificant variations in the shape of the male carapace (blue dots) and female carapace (red dots) of *I. nimboni*. PC1 and PC2 depict 29.87% and 25.29% of variance, respectively.

In contrast to the variations observed in the shape of the female pleon and major cheliped, PCA revealed no distinct separation in the shape of the carapace between males and females of *I. nimboni* (Fig. 6). Shape variation of carapace among individual crabs was unclear. Results of PCA are supported by both CVA and DFA (Table 2). The Procrustes distance of carapace between the sexes is 0.023 (*p* = 0.163), which revealed no statistical evidence for differences in the shape of the carapace between males and females of *I. nimboni*. Results of Hotelling’s *T*^2^ (DFA) indicate no significant differences in carapace shape by CVA and has strong taxonomic implications (Table 2).

**Figure 7:**
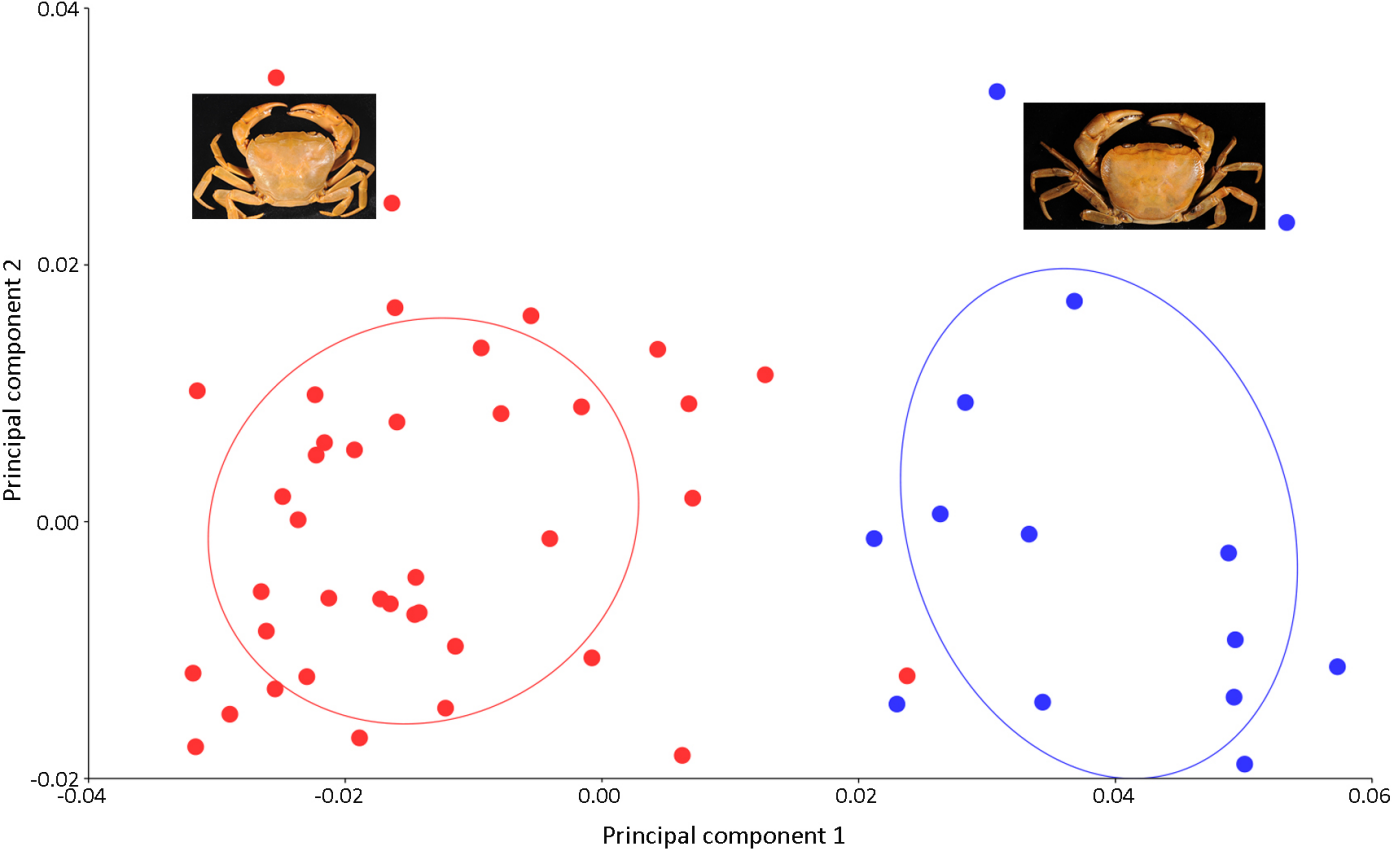
PCA showing significant difference in the shape of the carapace between *I. consobrinum* (red dots) and *I. nimboni* (blue dots). PC1 and PC2 depict 47.72% and 11.76% of variance, respectively.

Results of the multivariate analyses clearly show that variation in the shape of the carapace is insignificant and consistent across male and female crabs of *I. nimboni*. Therefore, the characteristic was selected for distinguishing *I. nimboni* from *I. consobrinum*. PCA revealed a distinct separation between the two species as far as the shape of the carapace is concerned (Fig. 7). Results of PCA are supported by both CVA and DFA (Table 3). The Procrustes distance of carapace between species is 0.053 (*p* < 0.0001), which provides statistical evidence for differences in the shape of the carapace between the two species. Results of Hotelling’s *T*^2^ (DFA) also support the results of PCA and CVA, which further strengthen the fact that the shape of carapace is taxonomically important (*T*^2^ = 858.32, *p* < 0.0001) (Table 3). These findings revealed the shape of the carapace to be a better characteristic for the species diagnosis in female freshwater crabs.

**Table 3:** Canonical variate analysis and discriminant function analysis indicating difference in the shape of the carapace between *Isolapotamon nimboni* and *Isolapotamon consobrinum*.

Character	Canonical variate analysis		Discriminant function analysis	
	Procrustes distance	*p*-Value	Hotelling’s *T*^2^	*p*-Value
Carapace	0.053	<0.0001	858.32	<0.0001

## Discussion

Our findings are in concordance with those studies that also showed significant variations in the shape of carapace, as in the case of 10 species of a marine genus (*Uca* Leach, 1814) from the Brazil coast (Hampton et al., 2014) and two species of freshwater crabs (*Zilchiopsis collastinensis*; Pretzmann, 1968 and *Trichodactylus borellianus*; Nobili, 1896) from the Paraná River of Argentina (Torres, Collins & Girl, 2014). Unlike the carapace, the pleon of freshwater crabs shows significant variation in shape during ontogeny. As far as the chela is concerned, regeneration has been proven to affect the size allometry and mean shape of the structure in species of *Uca* (Rosenberg, 2002). The same biological process could be occurring to a lesser degree in freshwater crabs. Therefore, we anticipated that one possible explanation for the result could be the selection of landmarks on the major chela, which was inconsistent due to the variability of the character itself, resulting from regeneration effects and deformity. Other studies have indicated that morphs, growth patterns (i.e., allopatric and sympatric), preservative, and digitizing method (e.g., scanner or camera) could influence the results of geometric morphometric analyses (Klingenberg, Barluenga & Meyer, 2003; Rufino, Abelló & Yule, 2006; Giri & Loy, 2008; Berbel-Filho, Jacobina & Martinez, 2013; Martinez, Berbel-Filho & Jacobina, 2013).

Our study indicates that the advantage of the choice of landmarks on the carapace over those on the pleon and chela is their relatively flat orientation, allowing consistency and ease in data preparation for geometric morphometric analysis. Nevertheless, Ng & Earl of Cranbrook (2014) demonstrated that morphology of fossilized chela can be used to distinguish the freshwater-dependent families of crabs such as Potamidae, Gecarcinucidae, and Sesarmidae. More importantly, chelipeds often constitute the major proportion of excavated freshwater crab fossils. This is very likely in localized areas where the crab fauna is well known.

Although the shape of the carapace can be used to statistically distinguish species, a linear two-dimensional measurement of the character cannot be established in the present study. Certainly, the conventional morphometric technique cannot be applied, and the shape analysis by geometric morphometric is proposed. It was noted that geometric morphometric analysis is inexpensive, as all the software used is available as freeware. In addition, the technique is easier and faster than molecular approaches. It is even suitable for amateurs.

Unstable characters are intrinsically unsuitable for diagnosis. Rather, the main challenge is in the paucity of characters, especially for females. Many characters have been proposed as useful traits for taxon (family, genus, and species) diagnosis, including carapace, third maxilliped with exopod, mandibular palp, male gonopods, thoracic sternites, endophragmal system, and frontal median triangle (Ng, 1988; Cumberlidge, 1999; Ng & Tan, 1999; Sternberg & Cumberlidge, 2003; Yeo & Ng, 2004; Reed & Cumberlidge, 2006; Ng, Guinot & Davie, 2008); the variability and instability of some structures remain debated. The instability of characters is more noticeable within the lower taxonomic ranks (i.e., genus and species) and among the taxa from different geographic regions (Ng & Tan, 1999; Brandis, Storch & Türkay, 2000; Yeo & Ng, 2004; Noël & Guinot, 2007). Carcinologists are more comfortable with the structure of the male gonopods, which is presumably the most stable and dependable feature for the diagnosis of freshwater crab species (Ng, 1988; Cumberlidge, 1999; Reed & Cumberlidge, 2006) even though gonopod morphology could be markedly variable, particularly within high-level monophyletic groups (Noël & Guinot, 2007). Moreover, new characteristics for species diagnosis of female freshwater crabs have not been reported.

## Conclusion

Considering the challenges in taxonomic and systematic studies of freshwater crabs, our study aims to highlight the difficulties in the current practices of identifying female crabs and provides better characteristics for their diagnosis. Conventional morphometrics (two-dimensional measurement) is fast and inexpensive, but unreliable at the level of accuracy. In contrast, molecular techniques time-consuming and can be expensive. The limitation of these approaches suggests other techniques should be explored. The present study attempts to apply the geometric morphometrics in identifying better characteristics for the diagnosis of female crabs of Sarawak. It is proposed that the shape of the carapace should be a reliable characteristic for the identification of female freshwater crabs using the geometric morphometric technique. The technique is inexpensive and relatively less time consuming, and the accuracy of the results are encouraging. The proposed technique is particularly useful where dissection to examine the gonopods is not feasible, as in the case of live or precious specimens.

## Supplemental Information

10.7717/peerj.6205/supp-1 Supplemental Information 1 Landmarks for geometric morphometric analysis. The tps files can only be viewed using tpsDig232 program, and should be opened along with the images.

## Additional Information and Declarations

### Field Study Permissions

The following information was supplied relating to field study approvals (i.e., approving body and any reference numbers): Research Permit was granted by Forest Department of Sarawak (Research permit No. 349/2012).

### Data Availability

The following information was supplied regarding data availability: Raw data is provided in the Supplemental Files.
