# Supplementary figures and images for: Geometric morphometric analysis in female freshwater crabs of Sarawak (Borneo) permits addressing taxonomy-related problems
Source: PeerJ. 2019 Feb 14;7:e6205. doi: 10.7717/peerj.6205 (PMC6378089; doi:10.7717/peerj.6205)

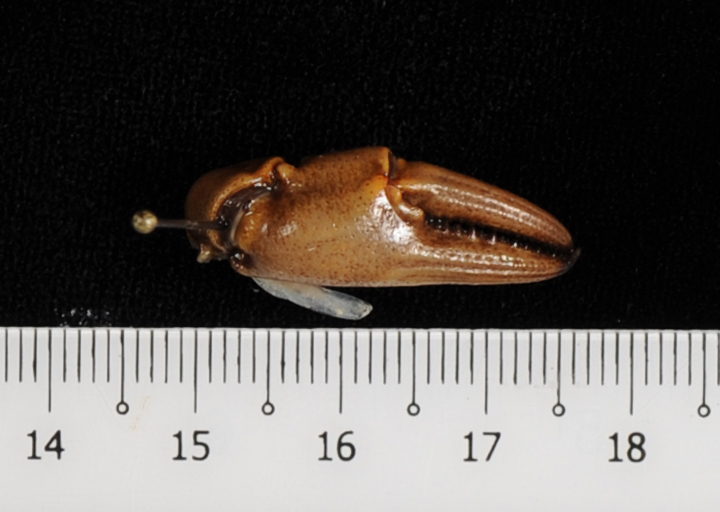

Supplement: Supplemental Information 1 [file peerj-07-6205-s001.zip › raw_materials/Inimboni_female_CH_724005.JPG]

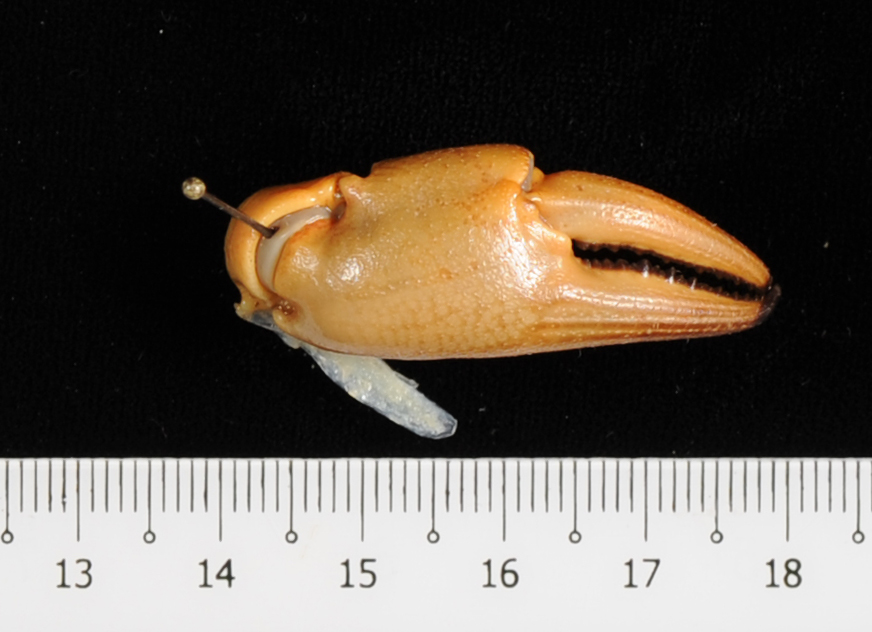

Supplement: Supplemental Information 1 [file peerj-07-6205-s001.zip › raw_materials/Inimboni_female_CH722801.JPG]

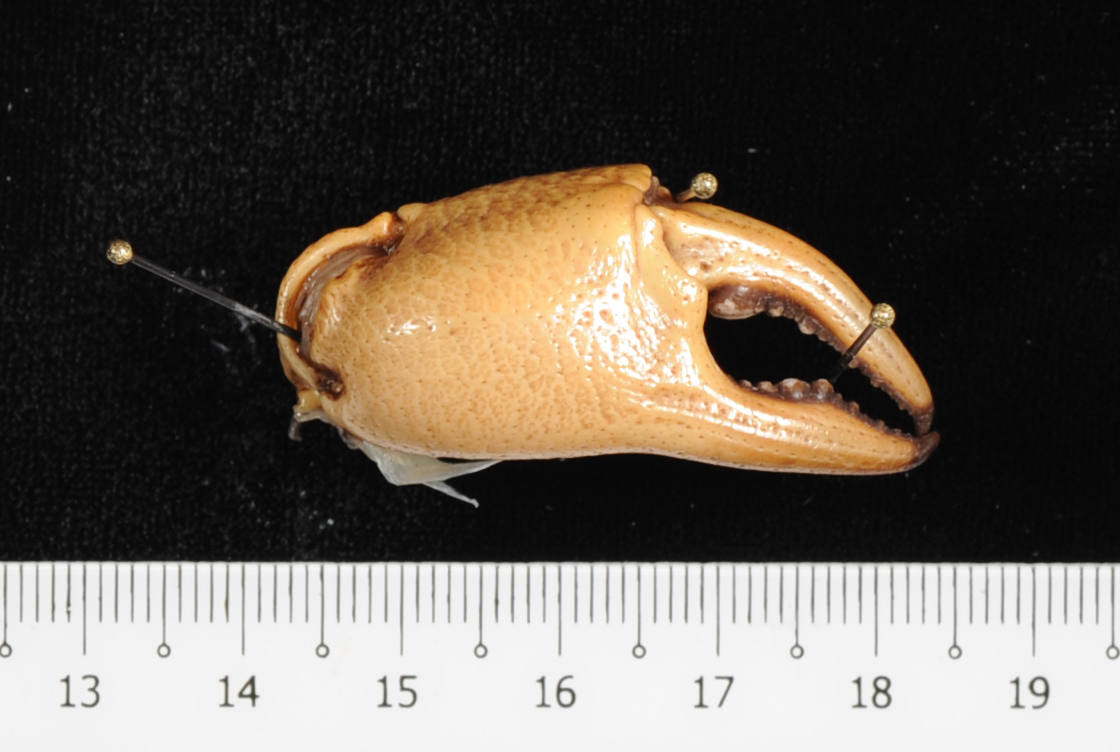

Supplement: Supplemental Information 1 [file peerj-07-6205-s001.zip › raw_materials/Inimboni_female_CH723102.JPG]

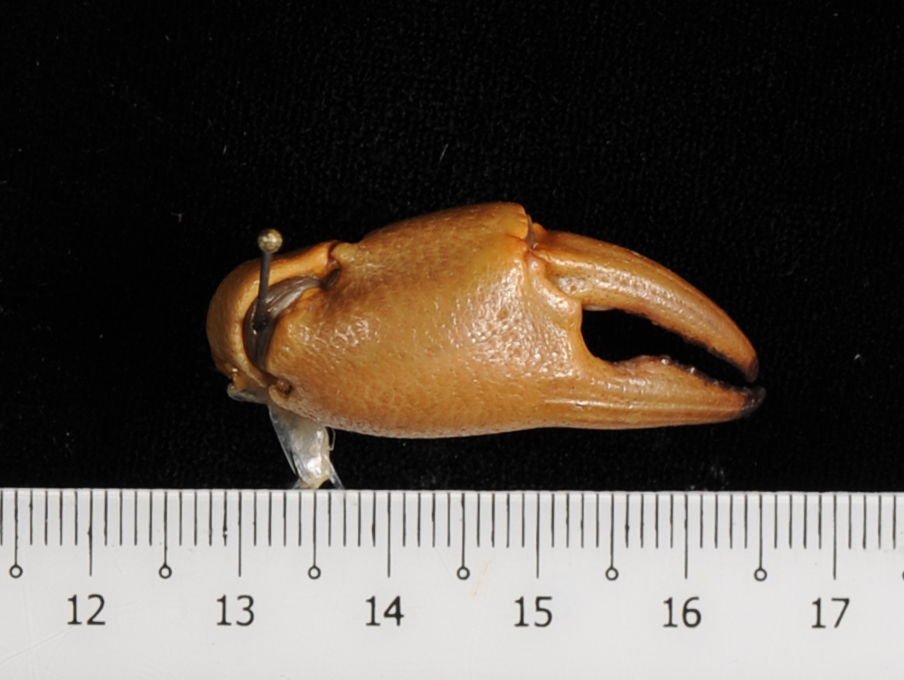

Supplement: Supplemental Information 1 [file peerj-07-6205-s001.zip › raw_materials/Inimboni_female_CH723403.JPG]

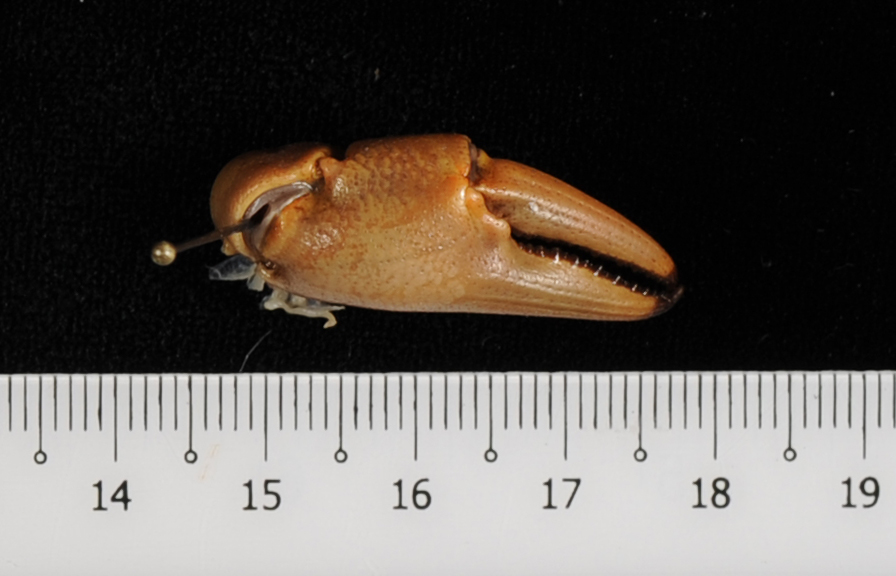

Supplement: Supplemental Information 1 [file peerj-07-6205-s001.zip › raw_materials/Inimboni_female_CH723804.JPG]

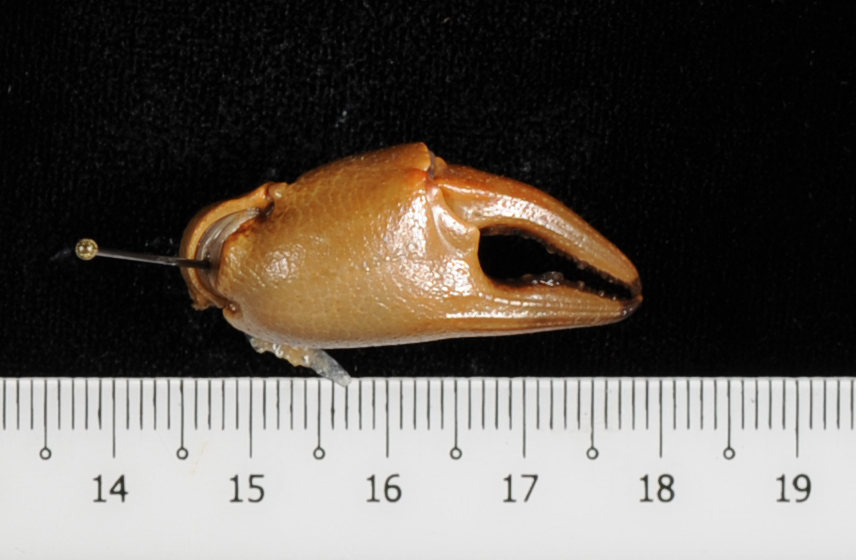

Supplement: Supplemental Information 1 [file peerj-07-6205-s001.zip › raw_materials/Inimboni_female_CH724006.JPG]

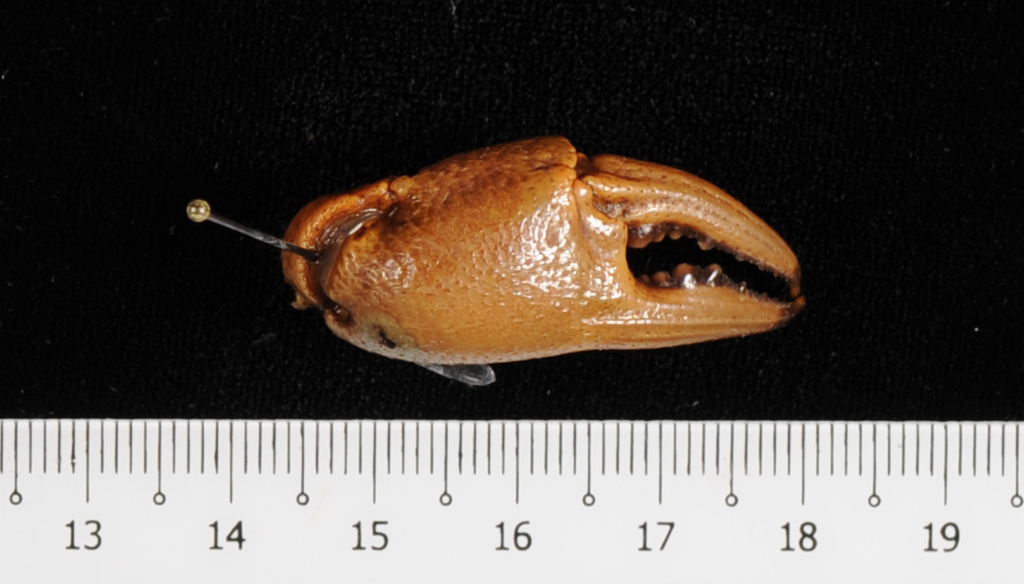

Supplement: Supplemental Information 1 [file peerj-07-6205-s001.zip › raw_materials/Inimboni_female_CH724607.JPG]

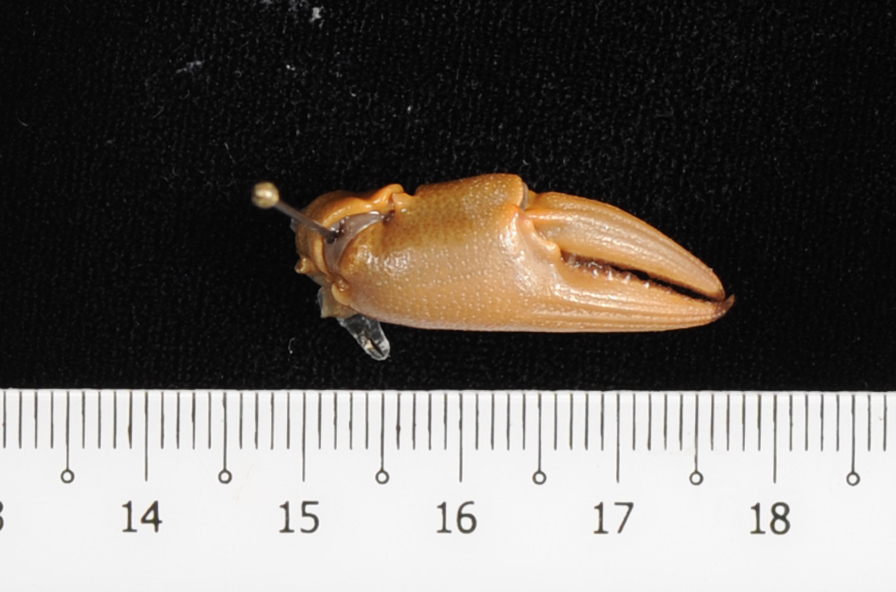

Supplement: Supplemental Information 1 [file peerj-07-6205-s001.zip › raw_materials/Inimboni_female_CH724908.JPG]

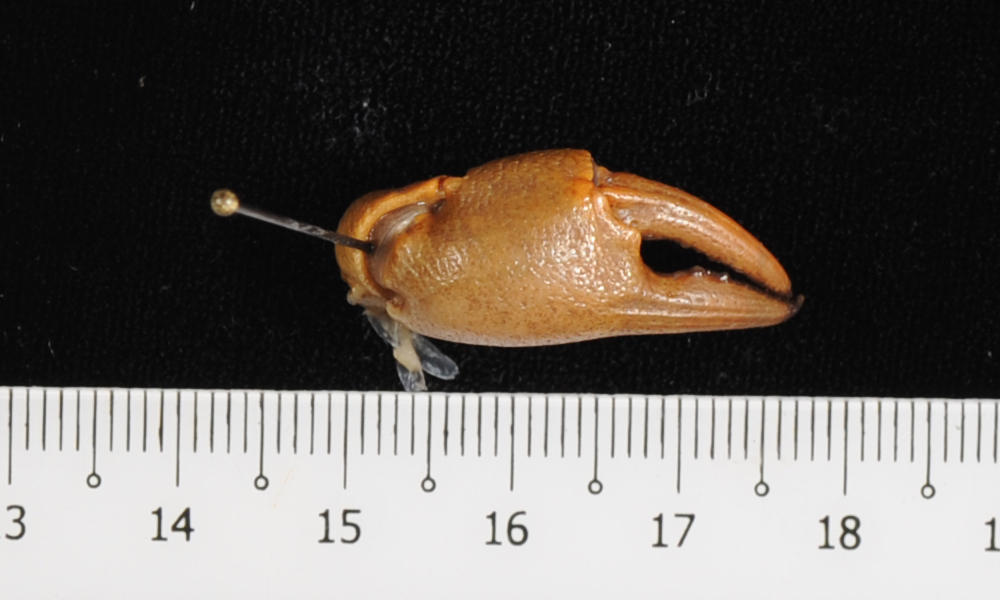

Supplement: Supplemental Information 1 [file peerj-07-6205-s001.zip › raw_materials/Inimboni_female_CH725209.JPG]

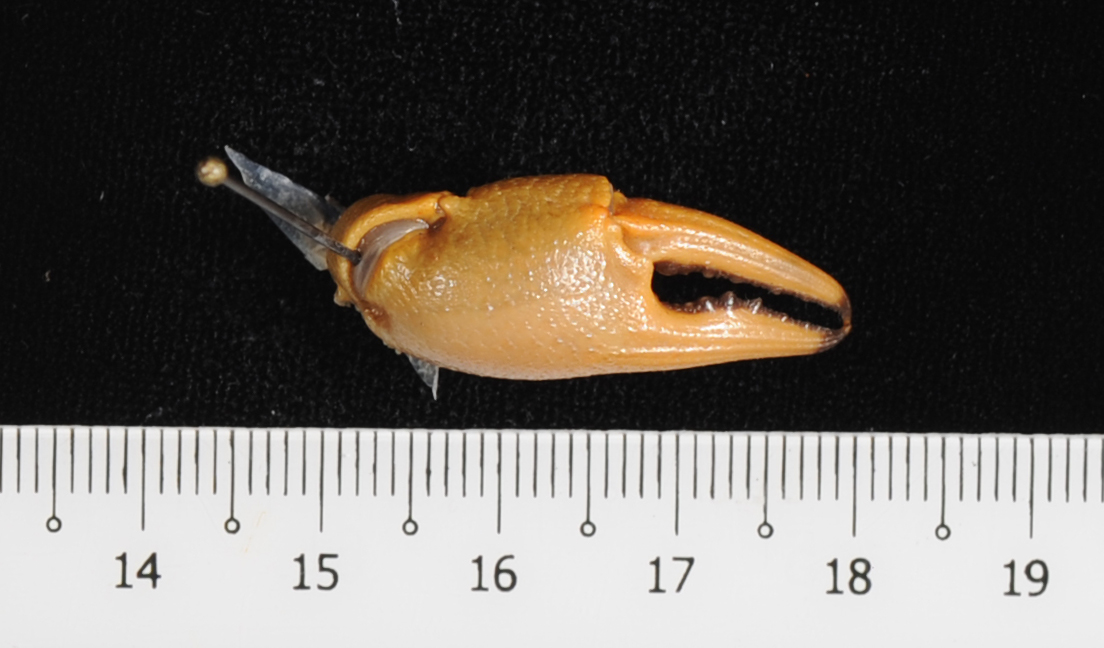

Supplement: Supplemental Information 1 [file peerj-07-6205-s001.zip › raw_materials/Inimboni_female_CH725510.JPG]

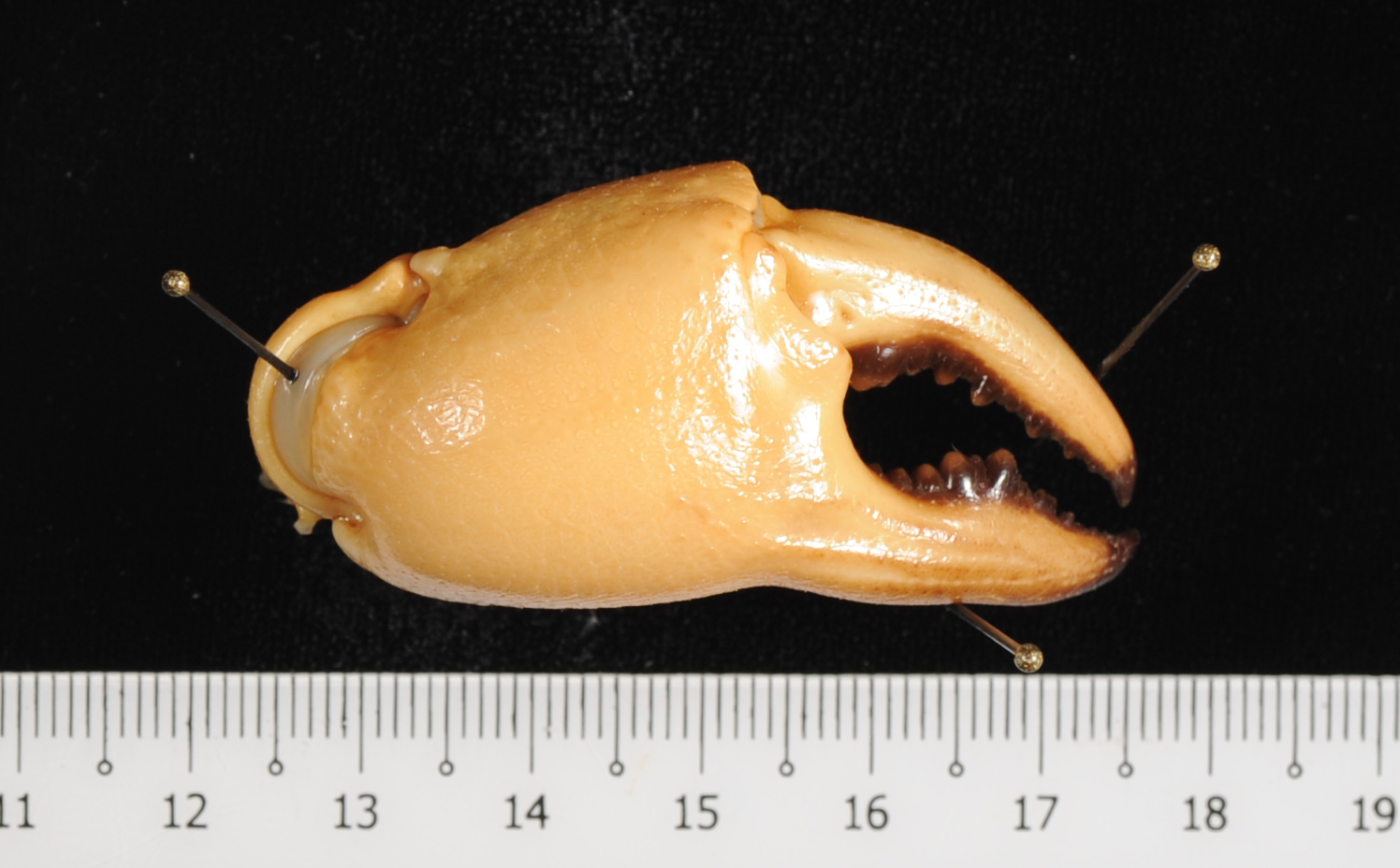

Supplement: Supplemental Information 1 [file peerj-07-6205-s001.zip › raw_materials/Inimboni_male_CH_7258_11.JPG]

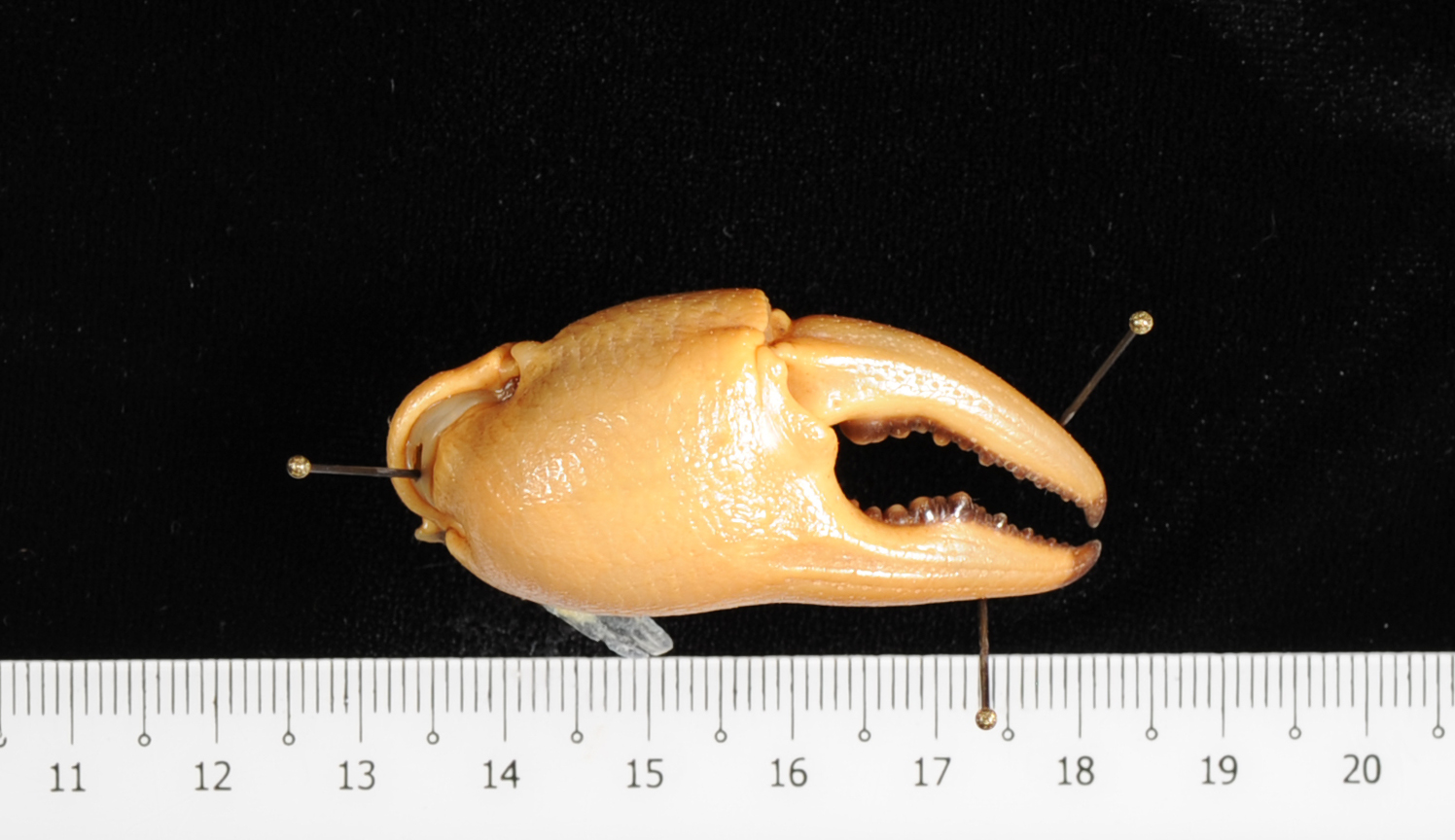

Supplement: Supplemental Information 1 [file peerj-07-6205-s001.zip › raw_materials/Inimboni_male_CH_7261_12.JPG]

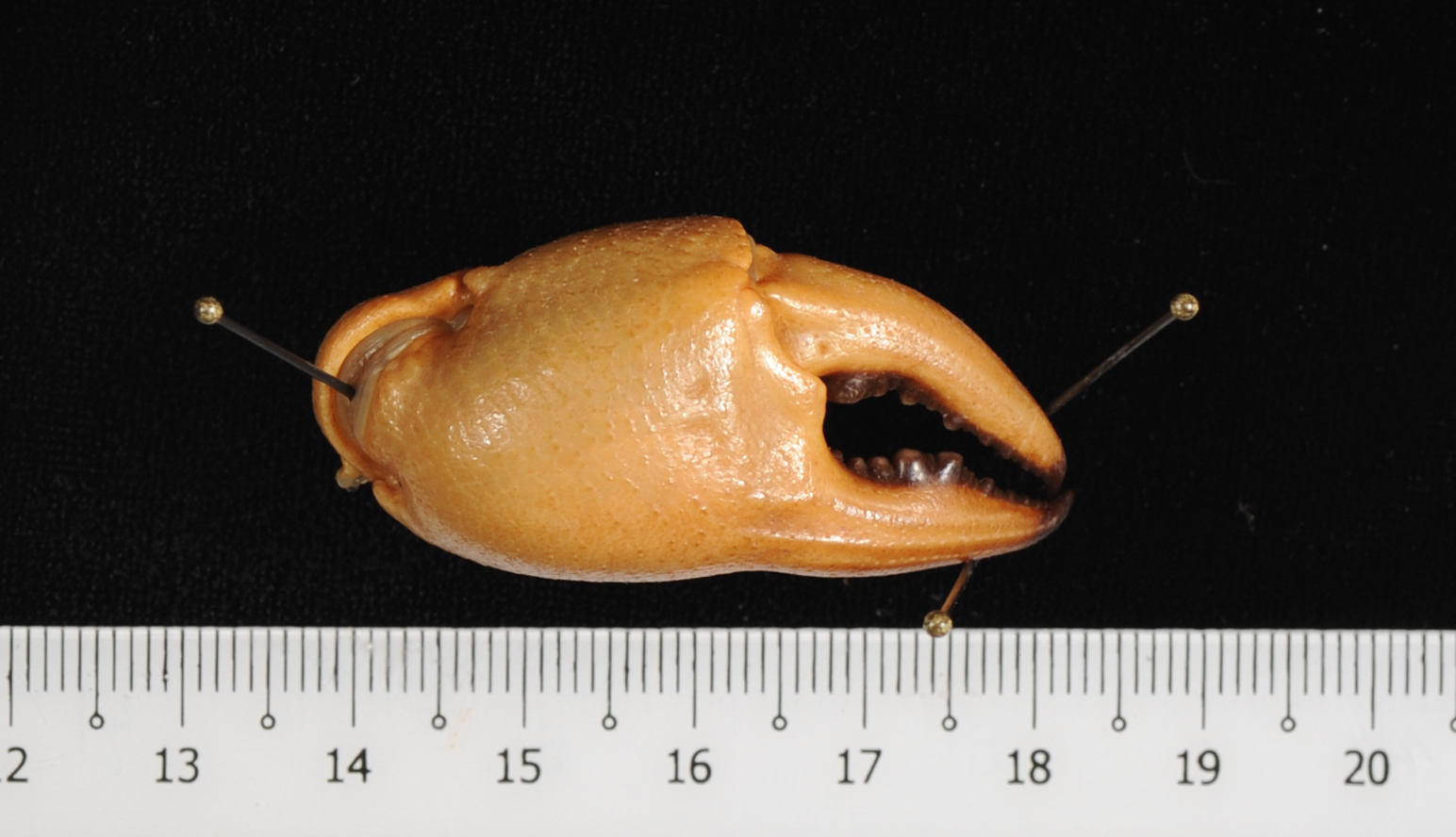

Supplement: Supplemental Information 1 [file peerj-07-6205-s001.zip › raw_materials/Inimboni_male_CH_7264_13.JPG]

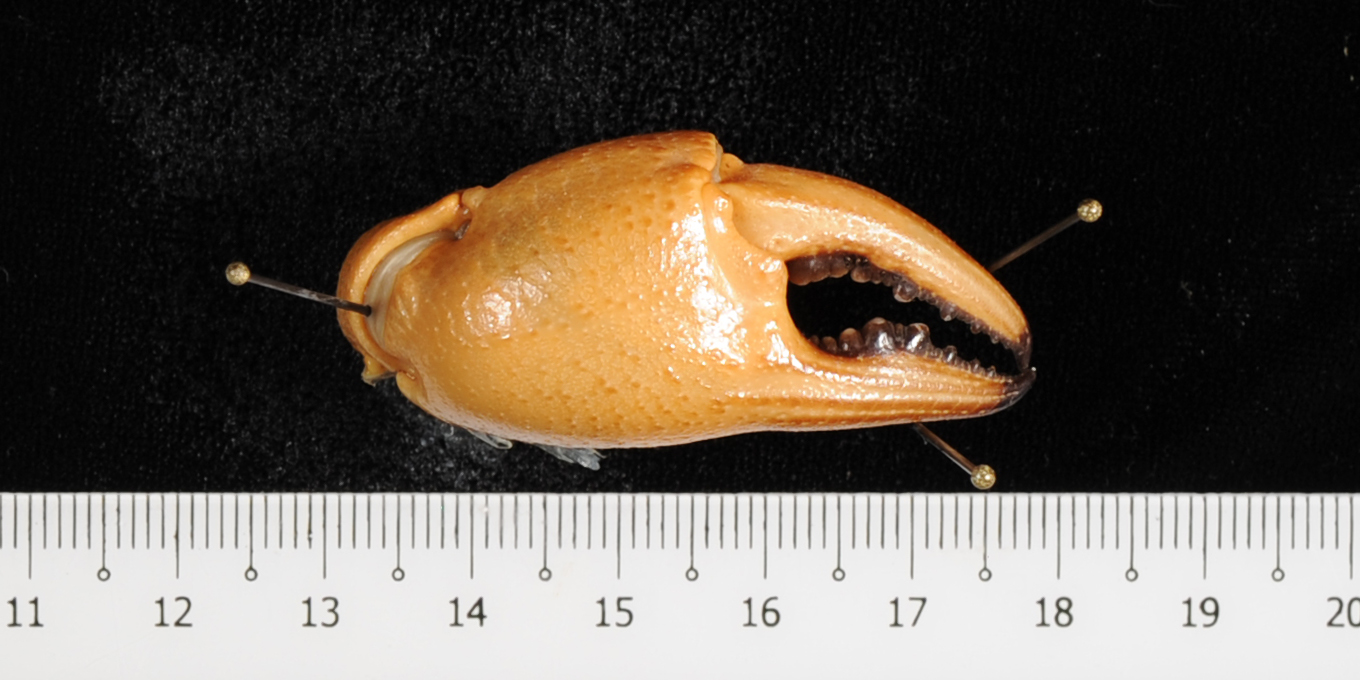

Supplement: Supplemental Information 1 [file peerj-07-6205-s001.zip › raw_materials/Inimboni_male_CH_7267_14.JPG]
